# Spinal Epidural Hematoma: A Case Series and the Current Paradigm

**DOI:** 10.7759/cureus.66756

**Published:** 2024-08-13

**Authors:** Ghim Hoe Neo, Marco Lizwan, Haobin Chen

**Affiliations:** 1 Orthopaedic Surgery, Sengkang General Hospital, Singapore, SGP; 2 General Surgery, Singapore General Hospital, Singapore, SGP

**Keywords:** weakness in limbs, traumatic spinal epidural hematoma, spontaneous spinal epidural hematoma, spinal epidural hematoma (seh), spinal cord

## Abstract

Spinal epidural hematoma (SEH) is a rare but serious condition characterized by the accumulation of blood in the spinal epidural space. As SEH progresses, it can result in permanent damage or paralysis if not treated promptly. We report three cases of SEH: one spontaneous and two traumatic. Timely diagnosis and intervention led to favorable outcomes, with significant neurological recovery in all cases. Minimizing the extent of laminectomy in evacuating the SEH reduced the likelihood of post-laminectomy kyphosis while avoiding the need for spinal instrumentation. More research is required to optimize the treatment protocols for SEH and further improve patient outcomes.

## Introduction

Spinal epidural hematoma (SEH) is a rare but serious condition that is characterized by the accumulation of blood in the spinal epidural space. SEH can result in acute spinal cord compression and eventually permanent damage or paralysis if left untreated. Several pathologies causing SEH have been described, and they can be broadly categorized into spontaneous and non-spontaneous types. Pathologies for spontaneous SEH include idiopathic causes, coagulopathy, vascular malformation, and pregnancy, whereas those for non-spontaneous SEH include trauma, medical procedures, and infections [1]. We report three cases of SEH in this series.

## Case presentation

Case 1

**Figure 1:**
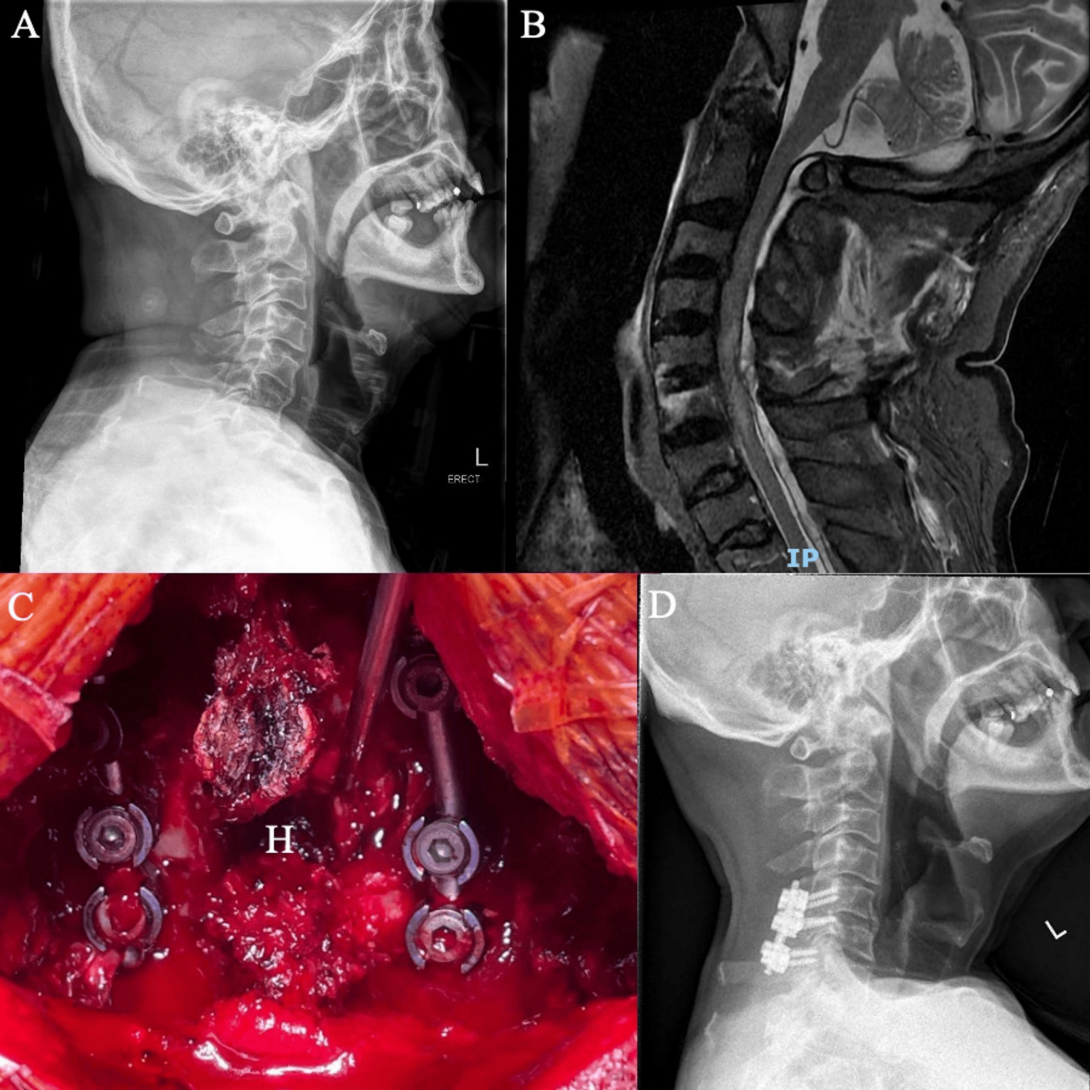
Case 1 (A) Lateral X-ray of the cervical spine showing minimally displaced vertebral body fractures involving the C5 and C6 levels. (B) MRI of the cervical spine (MRI-CS) showing a large epidural hematoma spanning C1 to T2 with cord edema from C4 to C6. (C) Intraoperative photograph with hematoma shown (labeled “H”). (D) Postoperative lateral XR cervical spine showing stable implants

A 73-year-old male presented to the Emergency Department with neck pain and upper limb weakness after falling off of a stationary motorcycle. Physical examination revealed tenderness over the C5/C6 region with motor deficits in the bilateral C6-T1 segment [Medical Research Council (MRC) grade 2]. His neurological examination was otherwise unremarkable. Initial imaging showed minimally displaced vertebral body fractures involving the C5 and C6 levels (Figure 1A), with a CT scan of the brain showing no intracranial pathology. The imaging showed no thoracic or lumbar spine involvement. The patient was admitted to the High Dependency Unit with a cervical collar. Subsequent MRI of the cervical spine (MRI-CS) showed a large epidural hematoma spanning C1 to T2, with cord edema from C4 to C6 (Figure 1B). 

The patient underwent posterior cervical decompression and instrumentation within 12 h from initial presentation to the Emergency Department. Evacuation of the hematoma was done through an extended C6 laminectomy with flushing via an infant feeding catheter passed cranially and caudally. Instrumentation of the C5-C7 levels with lateral mass screws was performed. Intraoperatively, there was a large, organized epidural hematoma over C5-C7 with extension both cranially and caudally (Figure 1C). His postoperative recovery was unremarkable. He reported improvement in upper limb strength on postoperative day (POD) two. The patient was transferred to the Rehabilitation Department on POD five. He was discharged three weeks postoperatively with documented improvement of upper limb power (ranging from MRC grade 3 to 4) in the bilateral C6-T1 levels. 

The patient was seen in a specialist outpatient clinic at one and four months postoperatively. Follow-up radiographs showed stable implants, and the patient reported ongoing improvement in neurological function (Figure 1D). His neurological findings at four months post-surgery were documented as MRC grade 5 in the entire right upper limb, grade 4 in the left C6-7 levels, and grade 3 in the left C7-T1 levels. 

Case 2

**Figure 2:**
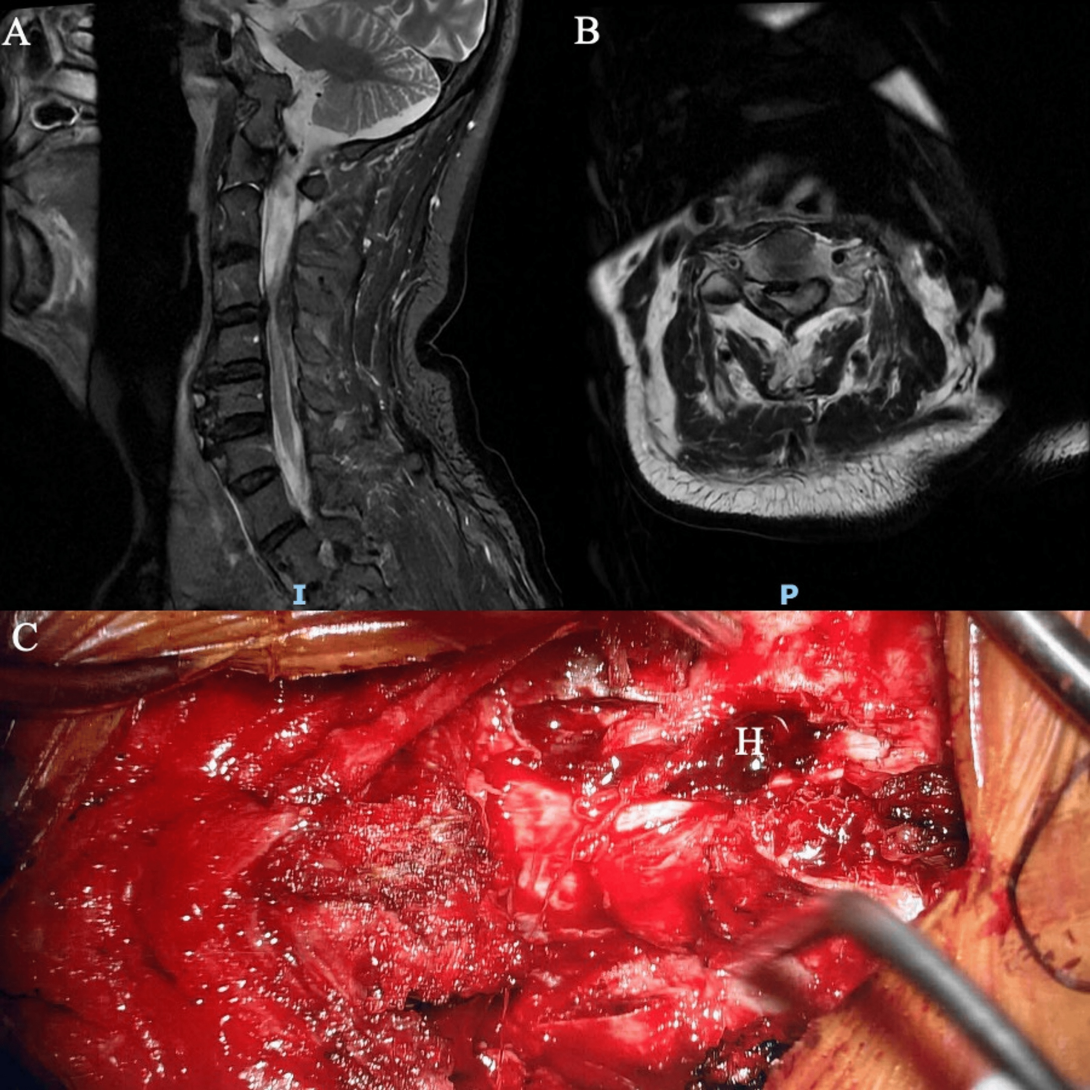
Case 2 MRI cervical spine (A) sagittal and (B) axial views showing right-sided focal epidural hematoma with cord edema spanning the C2-C4 levels. (C) Intraoperative photograph with hematoma shown (labeled “H”)

A 79-year-old male with a significant medical history of previous left lacunar infarct with full functional recovery (on lifelong aspirin) presented to the Emergency Department with right-sided upper and lower limb weakness with no precipitating events. Symptom onset was characterized by mild right-sided motor weakness, which progressed to complete right-sided hemiplegia (MRC grade 0). The sensation was preserved. Initial investigations showed no coagulopathy, and a CT scan of the brain showed no intracranial hemorrhage or infarct. The patient was admitted to the Acute Stroke Unit for possible stroke. A subsequent MRI of the brain showed no evidence of acute stroke. MRI-CS was subsequently performed, which showed a right-sided focal epidural hematoma with cord edema spanning the C2-C4 levels (Figures 2A-2B).

An urgent consult with the Department of Spinal Surgery was made overnight. The patient underwent posterior cervical decompression the following day. Evacuation of the hematoma was performed through a C3 laminectomy and right-sided C4 partial laminectomy with flushing via an infant feeding catheter passed cranially and caudally. Intraoperatively, there was an organized hematoma extending from the right C3-C4 levels (Figure 2C).

The patient’s postoperative recovery was uneventful. He reported slight improvement of right-sided motor function on POD two with flickers of movement. At two months postoperatively, his neurological function significantly improved (right upper limb MRC grade 3 and right lower limb MRC grade 4), and he was discharged to a community hospital for further rehabilitation.

Case 3

**Figure 3:**
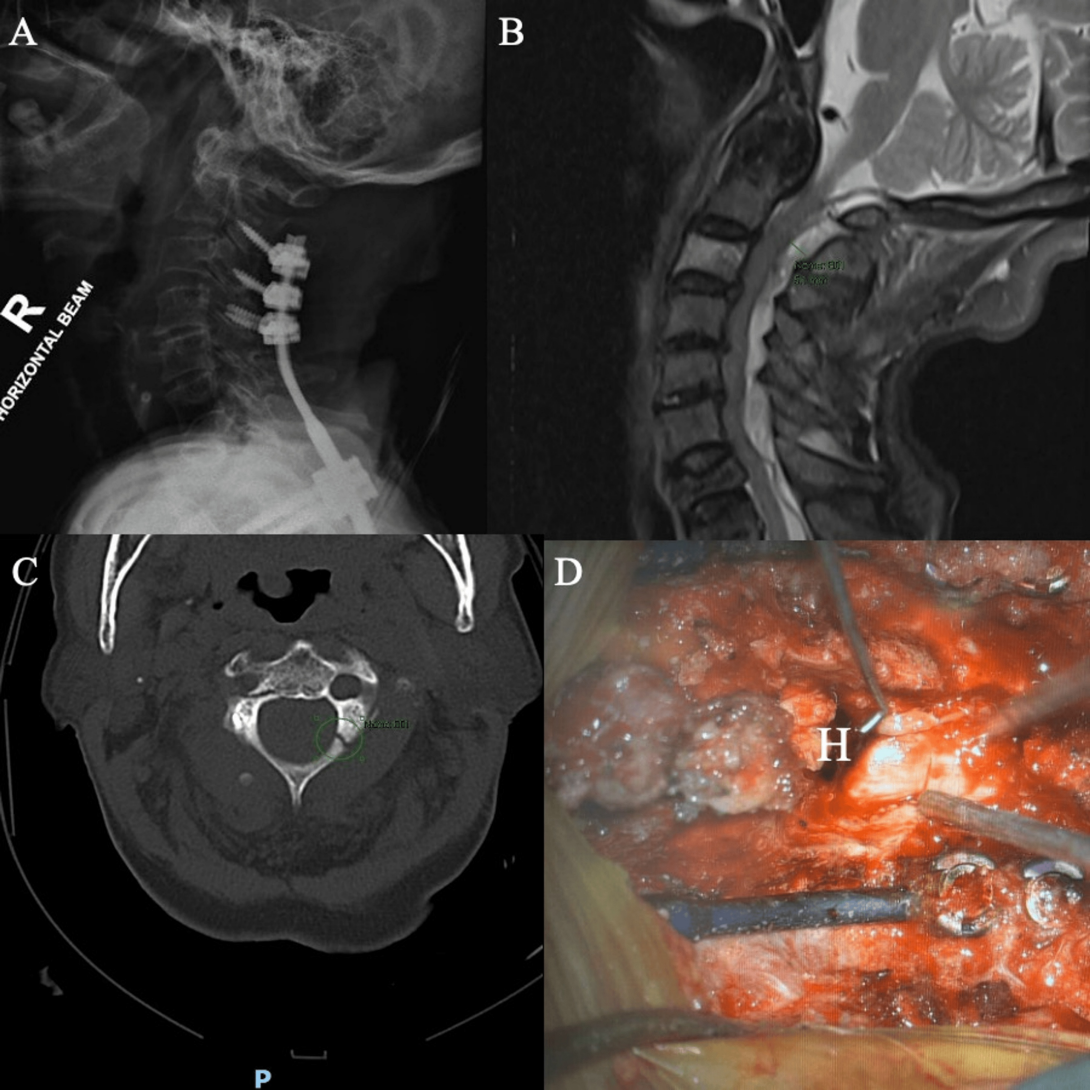
Case 3 (A) Lateral X-ray of the cervical spine showing C2-T4 posterior instrumentation for right C6/7 facet dislocation. (B) CT cervical spine showing minimally displaced C2 (atypical hangman) fracture with loosened left C2 screw. (C) MRI-CS showing an extensive epidural hematoma spanning from foramen magnum to T1 with cord edema at the C4-C6 levels and multi-level stenosis at the C3-C6 levels. (D) Intraoperative photograph with hematoma shown (labeled “H”)

An 81-year-old female with previous C2-T4 posterior instrumentation for right C6/7 facet dislocation (Figure 3A) presented to the Emergency Department with a scalp laceration after a fall resulting in head injury. Her neurological function was intact at the time of presentation. She noted numbness over the left upper and lower limbs two hours later with reduced motor movement over her entire left side. A CT scan of the brain showed no intracranial pathology, and a CT scan of the cervical spine showed a minimally displaced C2 (atypical hangman) fracture with a loosened left C2 screw (Figure 3B). Urgent MRI-CS was performed, which showed an extensive epidural hematoma spanning from the foramen magnum to the T1 level, with cord edema at the C4-C6 levels and multi-level stenosis at the C3-C6 levels (Figure 3C). Erect radiography showed no spondylolisthesis of the C2/3 level. Serial neurological examination showed decreased power of the bilateral upper limbs (MRC grade 3-4) and lower limbs (MRC grade 0).

The patient underwent posterior cervical decompression within 12 h of symptom onset. Evacuation of the hematoma was performed through an extended C3-C6 laminectomy with flushing via an infant feeding catheter passed cranially and caudally. Intraoperatively, there was a mixture of fresh and organized hematoma extending from the C2 to C7 levels (Figure 3D). Even though she was not on any anticoagulation treatment, significant oozing was noted intraoperatively, necessitating meticulous hemostasis using multiple hemostatic agents. Postoperatively, the patient admitted to using traditional Chinese supplements, which might have contributed to the formation of the SEH.

The patient’s postoperative recovery was unremarkable. She reported improvement of bilateral upper limb motor function on POD one with some flickers of movement over the right lower limb, while her left lower limb remained flaccid. There was significant neurological recovery at one month postoperatively (right upper limb MRC grade 4, right lower limb MRC grade 5, left upper and lower limb MRC grade 3), and she was able to ambulate with walking aids.

## Discussion

SEHs are space-occupying lesions resulting from the accumulation of blood in the vertebral epidural space, which requires prompt diagnosis and treatment to prevent morbidity and mortality. It is a relatively rare condition and can be spontaneous or caused by trauma. The sudden onset of severe back pain is a hallmark symptom of SEH, which is often accompanied by radicular pain that follows the distribution of affected nerve roots [2]. Neurological deficits can progress rapidly to motor weakness, sensory loss, and autonomic dysfunction. Furthermore, the level and severity of symptoms will depend on the location and size of the hematoma. MRI remains the gold standard for diagnosing SEH, as it can provide detailed images of the hematoma and assess the degree of spinal cord compression. Where MRI is not available, CT scan can be used in emergency settings [3]. Despite the use of imaging, clinical examination remains key to assessing the extent of spinal cord and nerve root involvement.

The mainstay of treatment for symptomatic SEH, especially in the context of progressive neurological deficits, is surgical intervention (e.g., laminectomy or hemilaminectomy) to evacuate the hematoma and decompress the spinal cord [4]. To achieve better neurological outcomes, early surgical intervention (ideally within 12-24 h of symptom onset) is recommended [5]. In cases of small asymptomatic hematomas, conservative management such as bed rest, pain management, and close neurological monitoring can be considered [6]. Prompt diagnosis and treatment are crucial to prevent permanent neurological deficits. Depending on the initial severity of SEH and the timeliness of intervention, some patients may have residual deficits, whereas others may achieve complete recovery.

Surgical technique

In all of our patients, evacuation of the hematoma was performed using an infant feeding catheter by passing the catheter through the laminectomy window both cranially and caudally followed by flushing with saline. This technique allows for the evacuation of large hematomas while avoiding the need to perform extensive laminectomy to achieve adequate evacuation. With a shorter laminectomy, the stability of the spine can be preserved. Careful preoperative evaluation of MRI scans must be done to ensure that the catheter is not being passed through stenotic areas, which could result in dura injuries. Intraoperative neuromonitoring remained stable throughout, and there were no suggestions of dura injuries sustained during the procedure in any of the three cases.

For our first patient, an extensive hematoma was formed even though only focal fractures involving the C5 and C6 vertebral bodies were sustained, with minimal displacement. The patient was not on any anticoagulant, and their initial workup did not suggest any coagulopathy. Instrumentation of the C5-C7 levels was performed in addition to the decompression and hematoma evacuation due to the involved fractures, and preoperative imaging found pre-existing segmental cervical spine kyphosis (Figure 1A). Clinical neurological improvement was observed as early as POD two.

In our third patient, a longer segment of laminectomy was performed, as an MRI scan revealed multi-level stenosis in the involved segments, rendering it unsafe to pass the infant feeding catheter for flushing of the hematoma with a short-segment laminectomy. Evacuation of the hematoma was performed through an extended C3-C6 laminectomy. Flushing via an infant feeding catheter passed cranially and caudally was still done to minimize the extent of laminectomy required, were the infant feeding catheter not used.

Diagnostic mimic

In our second case, the patient presented with symptoms mimicking those of acute stroke in the absence of trauma, which resulted in delayed surgical intervention. Spontaneous SEH (sSEH) remains rare, with an estimated incidence of 1 in 1,000,000 per year globally [7]. However, sSEH is a devastating condition that can deteriorate rapidly, resulting in neurological deficits with poor functional outcomes. Risk factors for sSEH are not well established and suggested associations include arteriovenous malformations, anticoagulation use, underlying coagulopathy, and vertebral hemangiomas, with 40-60% of cases demonstrating no identifiable risk factors for the hemorrhage [7]. Therefore, clinicians must maintain a high index of suspicion when handling patients presenting with stroke-like symptoms, especially for those with other associated risk factors. Our second patient was on aspirin for his previous lacunar stroke with no other identifiable risk factors.

Importance of timely intervention

In our third case, the patient initially presented with only a scalp laceration and intact neurological findings. Her symptoms only started a few hours later. As the patient was physically in the hospital, the time for imaging and surgery was minimal. Despite her poor preoperative neurological status, she exhibited significant neurological improvement, possibly due to the short time to surgery from the onset of symptoms.

Although studies have shown conflicting data regarding the recommended time of surgical intervention for SEH, the consensus is for emergent or at least urgent surgical intervention to be done promptly [5,8,9]. In all of our three cases, surgical intervention was achieved within 12 h of diagnosis, with two out of three cases operated on within 12 h of symptom onset. For cases 1-3, there was significant neurological improvement at four months, two months, and one month postoperatively, respectively. Preoperative neurological status remains the most important prognostic indicator for long-term outcomes [1]. A multidisciplinary team approach with early referral to the rehabilitation medical team is crucial for such patients with spinal cord injury, and this collaboration is well-established in our center.

## Conclusions

SEH is a medical emergency that requires a high index of clinical suspicion from clinicians. Timely diagnosis and intervention are critical for favorable neurological outcomes. In our cases, the use of infant feeding catheters allowed for the evacuation of large hematomas while avoiding the need for performing an extensive laminectomy to achieve adequate evacuation. Further research is required to optimize treatment protocols and improve patient outcomes in SEH.
